# Evaluation of the Anticancer Activity of a Bile Acid-Dihydroartemisinin Hybrid Ursodeoxycholic-Dihydroartemisinin in Hepatocellular Carcinoma Cells

**DOI:** 10.3389/fphar.2020.599067

**Published:** 2020-11-10

**Authors:** Tzu-En Huang, Yi-Ning Deng, Jui-Ling Hsu, Wohn-Jenn Leu, Elena Marchesi, Massimo L. Capobianco, Paolo Marchetti, Maria Luisa Navacchia, Jih-Hwa Guh, Daniela Perrone, Lih-Ching Hsu

**Affiliations:** ^1^ School of Pharmacy, National Taiwan University, Taipei, Taiwan; ^2^ Department of Chemical and Pharmaceutical Sciences, University of Ferrara, Ferrara, Italy; ^3^ Institute of Organic Synthesis and Photoreactivity, National Research Council, Bologna, Italy

**Keywords:** hepatocellular carcinoma, bile acid-dihydroartemisinin hybrid, G0/G1 arrest, apoptosis, reactive oxygen species, mitochondrial membrane potential loss

## Abstract

Hepatocellular carcinoma (HCC) is the most common primary liver malignancy in adults and accounts for 85–90% of all primary liver cancer. Based on the estimation by the International Agency for Research on Cancer in 2018, liver cancer is the fourth leading cause of cancer death globally. Dihydroartemisinin (DHA), the main active metabolite of artemisinin derivatives, is a well-known drug for the treatment of malaria. Previous studies have demonstrated that DHA exhibits antitumor effects toward a variety of human cancers and has a potential for repurposing as an anticancer drug. However, its short half-life is a concern and may limit the application in cancer therapy. We have reported that UDC-DHA, a hybrid of bile acid ursodeoxycholic acid (UDCA) and DHA, is ∼12 times more potent than DHA against a HCC cell line HepG2. In this study, we found that UDC-DHA was also effective against another HCC cell line Huh-7 with an IC_50_ of 2.16 μM, which was 18.5-fold better than DHA with an IC_50_ of 39.96 μM. UDC-DHA was much more potent than the combination of DHA and UDCA at 1:1 molar ratio, suggesting that the covalent linkage rather than a synergism between UDCA and DHA is critical for enhancing DHA potency in HepG2 cells. Importantly, UDC-DHA was much less toxic to normal cells than DHA. UDC-DHA induced G0/G1 arrest and apoptosis. Both DHA and UDC-DHA significantly elevated cellular reactive oxygen species generation but with different magnitude and timing in HepG2 cells; whereas only DHA but not UDC-DHA induced reactive oxygen species in Huh-7 cells. Depolarization of mitochondrial membrane potential was detected in both HepG2 and Huh-7 cells and may contribute to the anticancer effect of DHA and UDC-DHA. Furthermore, UDC-DHA was much more stable than DHA based on activity assays and high performance liquid chromatography-MS/MS analysis. In conclusion, UDC-DHA and DHA may exert anticancer actions via similar mechanisms but a much lower concentration of UDC-DHA was required, which could be attributed to a better stability of UDC-DHA. Thus, UDC-DHA could be a better drug candidate than DHA against HCC and further investigation is warranted.

## Introduction

Primary liver cancer includes hepatocellular carcinoma (HCC), intrahepatic cholangiocarcinoma, and other rare types. Among these types, HCC is the most common form of primary liver malignancy in adults, accounting for 85–90% of all primary liver cancers (Ozakyol, 2017). According to the estimation by the International Agency for Research on Cancer in 2018, liver cancer is the sixth most frequently diagnosed cancer and the fourth leading cause of cancer death for both sexes globally. Furthermore, it is the fifth most common cancer and the second leading cause of cancer death in men (Bray et al., 2018). Early-stage liver cancer can be treated with surgical approaches including liver resection and liver transplantation, and the 5-year survival rate is in the range of 60–70%. Unfortunately, most patients are diagnosed at advanced stages and no longer suitable to be treated with surgical approaches. These patients have to be treated with nonsurgical approaches, such as transarterial chemoembolization and transarterial radiation, or systemic approaches including targeted therapy, immunotherapy and chemotherapy; however, the survival rate of late-stage liver cancer remains low (Liu et al., 2015; Balogh et al., 2016; Ozakyol, 2017). Therefore, there is an urgent need for more effective treatments.

**FIGURE 1:**
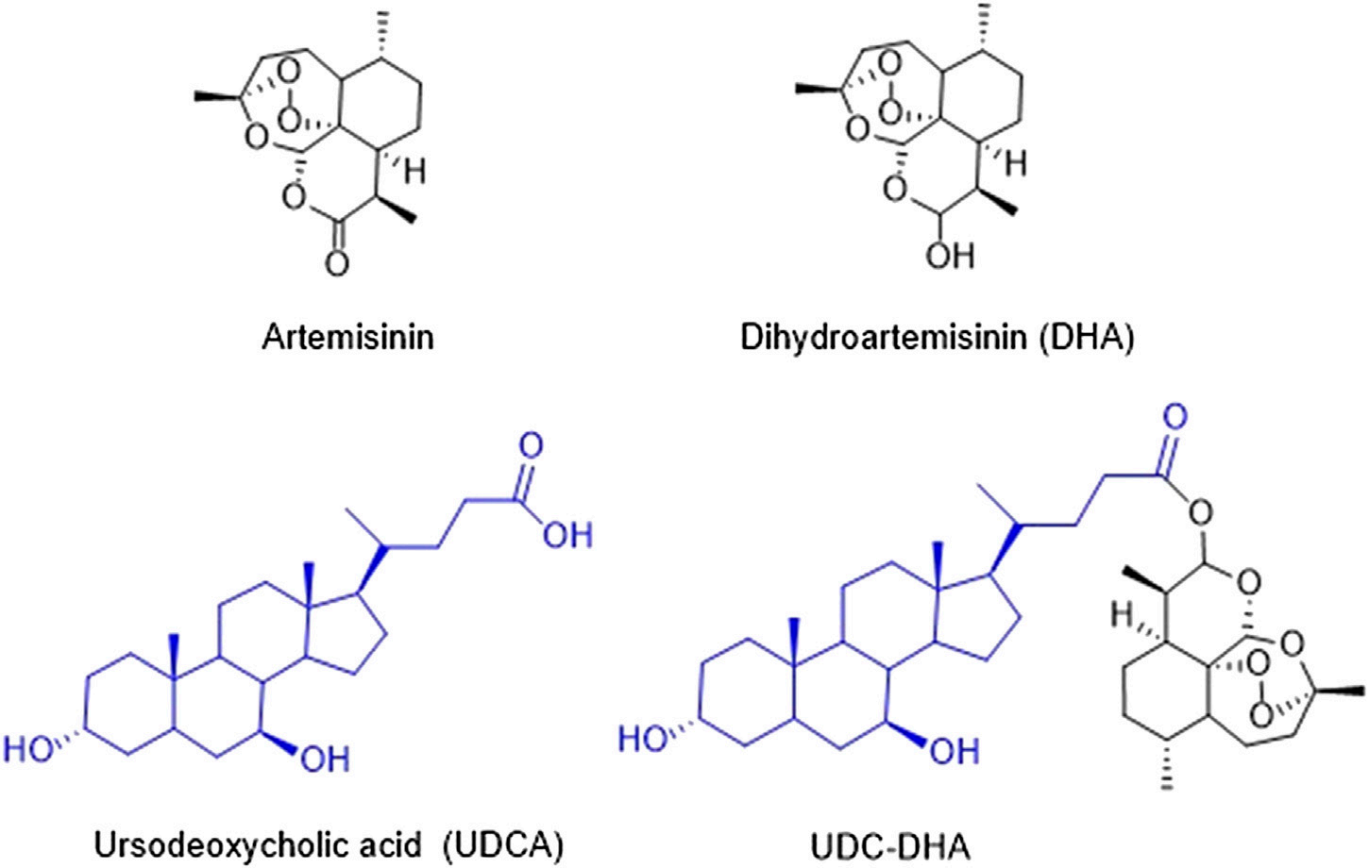
Chemical structures of artemisinin, DHA, UDCA, and UDC-DHA.

The Chinese herb *Qinghao* (*Artemisia annua*) has been used in traditional Chinese medicine for thousands of years for the treatment of fevers and chills. Dr. Youyou Tu’s research team identified artemisinin from *Artemisia annua* in 1972 as an effective antimalarial component which is a sesquiterpene lactone containing an endoperoxide bridge (Tu, 2011). Artemisinin (Figure 1) and its derivatives have become the standard therapy for malaria. In spite of the effectiveness against malaria, artemisinin derivatives are eliminated rapidly with a half-life of less than 1 h; therefore, multiple doses have to be administered each day. The WHO has recommended artemisinin-based combination therapies as the best treatment for malaria, combining an artemisinin derivative with another drug with a long half-life (Nosten and White, 2007).

Dihydroartemisinin (DHA) (Figure 1), the reduced lactol derivative of artemisinin, is more stable and ten times more potent than artemisinin (Tu, 2011). Furthermore, the hydroxyl group in DHA provides an opportunity of generating artemisinin derivatives through esterification. DHA is also the main active metabolite of artemisinin derivatives. Previous studies have shown that DHA exhibits anticancer activity toward a wide range of human cancers, including breast (Mao et al., 2013; Feng et al., 2016), leukemia (Lu et al., 2008; Wang et al., 2012), liver (Hou et al., 2008; Zhang et al., 2012; Qin et al., 2015), lung (Liao et al., 2014; Jiang et al., 2016), and pancreatic cancer (Li et al., 2016). It has been reported that DHA induces the generation of reactive oxygen species (ROS), further causes the depolarization of mitochondrial membrane potential (MMP) and ultimately leads to apoptosis (Hou et al., 2008). Other possible mechanisms have also been proposed, including cell cycle arrest, autophagy, ferroptosis, and DNA damage (Efferth, 2017; Wong et al., 2017). Although DHA exerts anticancer activity, the cytotoxic effect against cancer cells remains low partly due to its short half-life. Thus, several research groups have developed a series of DHA hybrids aiming to improve antitumor activity as well as stability (Smit et al., 2015; Xu et al., 2016; Yu et al., 2018).

Molecular hybridization is a widely used strategy to discover new active compounds. Bile acids (BAs), a group of acidic steroids, are synthesized from cholesterol in the liver. The enterohepatic circulation of bile acids is a very efficient recycling route in human body. Therefore, the bile acid transport system can be exploited for the design of drug delivery systems or prodrugs to improve intestinal absorption and metabolic stability, and even to target drugs to specific organs or sustain the release of active drugs at therapeutic concentrations (Sievänen, 2007; Faustino, et al., 2016). It has been reported that some BAs may be toxic toward cancer cells. Moreover, BAs may disturb or even damage the structure of cell membrane (Faustino, et al., 2016). However, the cytotoxicity of BAs is relatively low with IC_50_ greater than 100 μM (Horowitz et al., 2007; Faustino, et al., 2016). Thus, novel BA derivatives have been synthesized and exhibit potent anticancer effect toward several cancer cell lines (Faustino, et al., 2016).

We have synthesized a series of bile acid-dihydroartemisinin (BA-DHA) hybrids and evaluated the anticancer activity in a HCC cell line HepG2 (Marchesi et al., 2019). In this study, we further determined the activity of BA-DHA hybrids in another HCC cell line Huh-7. UDC-DHA, a hybrid of a bile acid ursodeoxycholic acid (UDCA) and DHA (Figure 1), which was 10–20 times more potent than DHA in HepG2 and Huh-7 cells, was chosen for the investigation of the mechanisms of action.

## Materials and Methods

### Chemicals

DHA was purchased from Carbosynth (Compton, Berkshire, United Kingdom) (purity ≥98%), and BAs were kindly provided by ICE SpA (Reggio Emilia, Italy). BA-DHA hybrids were synthesized as described previously (Marchesi et al., 2019). 3-(4,5-Dimethylthiazol-2-yl)-2,5-diphenyltetrazolium bromide (MTT), propidium iodide (PI), and JC-1 dye were obtained from Invitrogen Life Technologies (Carlsbad, CA). Sulforhodamine B (SRB) and 2′,7′-dichlorodihydrofluorescein diacetate (DCFH-DA) were purchased from Sigma-Aldrich (St. Louis, MO). N-acetyl-L-cysteine (NAC) was purchased from MedChemExpress (Monmouth Junction, NJ). Stock solutions of DHA, UDCA, UDC-DHA and DCFH-DA were prepared in DMSO. MTT was dissolved in phosphate-buffered saline (5 mg/ml), 0.4% (w/v) SRB solution was prepared in 1% acetic acid, and NAC was dissolved in ddH2O.

### Cell Culture

Human HCC cell line HepG2 was obtained from the American Type Culture Collection (ATCC, Manassas, VA) and cultured in low-glucose DMEM supplemented with 10% FBS, 2 mM L-glutamine, and antibiotics including 100 units/ml penicillin, 100 μg/ml streptomycin, and 0.25 μg/ml amphotericin B. Another HCC cell line Huh-7 was obtained from Japanese Collection of Research Bioresources (JCRB, Ibaraki, Osaka, Japan) and cultured in high-glucose DMEM supplemented with 10% FBS, 2 mM L-glutamine, non-essential amino acids and antibiotics. Primary normal human dermal fibroblast (NHDF, p2) cells (C-12302) were purchased from PromoCell (Heidelberg, Germany) and cultured in PromoCell Fibroblast Growth Medium (C-23020) according to the manufacturer’s instructions. Cells were maintained at 37°C in a humidified 5% CO_2_ atmosphere.

### Cell Viability Assay and Growth Inhibition Assay

Cell viability was determined by the MTT assay. HCC and NHDF cells were seeded in 96-well plates at 5 × 10^3^ cells/well and 3 × 10^3^ cells/well, respectively. After overnight culture, cells were treated with indicated concentrations of compounds in culture medium for 24–72 h, followed by the MTT assay. Absorbance was measured at 570 nm with 690 nm as a reference wavelength using a SpectraMax Paradigm Multi-Mode Microplate Detection Platform (Molecular Devices, San Jose, CA). Cells incubated with DMSO served as the vehicle control, and others were normalized with the control. Cell growth inhibition was determined by the SRB assay. HepG2 cells were seeded in 96-well plates (5 × 10^3^ cells/well) and treated for 72 h and then subjected to the SRB assay as described (Lai et al., 2016) to determine cell growth inhibition. Absorbance was measured at 515 nm and growth inhibition (%) was calculated.

### Cell Cycle Analysis

Cells were seeded in 6-well or 12-well plates (1 × 10^5^ cells/well) and treated with indicated concentrations of compounds in culture medium for 24 and 48 h. Cells were then harvested by trypsinization, fixed overnight in 70% ethanol at −20°C, followed by propidium iodide (PI) staining and flow cytometric analysis using FACSCalibur (BD Biosciences, San Jose, CA). At least 10,000 cells were analyzed for each sample by FlowJo software (Tree Star, Ashland, OR).

### Caspase-Glo 3/7 Activity Assay

Cells were seeded in 96-wells (2.5 or 5 × 10^3^ cells/well) and treated with indicated concentrations of compounds for 72 h. Cells were then subjected to the Caspase-Glo 3/7 assay according to the manufacturer’s instructions (Promega, Madison, WI) and data was normalized with the cell number.

### Western Blotting

After drug treatment, cells were harvested and lysed in SDS-sample buffer. Cell lysates were subjected to 10 or 12% SDS-PAGE and Western blot analysis as previously described (Li et al., 2019). Primary antibodies used were PARP (BD Biosciences, San Jose, CA), caspase-3 (Cell Signaling Technology, Danvers, MA) and γ-tubulin (Sigma-Aldrich, St. Louis, MO). HRP-conjugated anti-mouse and anti-rabbit were used as secondary antibodies. Images were acquired and quantified using the ChemiDoc XRS system and Image Lab software (Bio-Rad, Hercules, CA).

### Measurement of Reactive Oxygen Species

Cells were seeded overnight in 12-well (1 × 10^5^ cells/well) and treated with indicated concentrations of compounds in culture medium with or without an antioxidant and ROS scavenger NAC at a final concentration of 2 mM for various time periods. DCFH-DA at a final concentration of 10 μM was added to the cells 30 min before the termination of the incubation period at 37°C. Cells were harvested by trypsinization, resuspended in cold PBS, subjected to flow cytometric analysis by FACSCalibur and data were analyzed by FlowJo software. Cells with ROS production were quantified.

### Measurement of Mitochondrial Membrane Potential

Cells were seeded overnight in 6-well plates (1–2 × 10^5^ cells/well), treated with indicated concentrations of compounds in culture medium with or without 2 mM of NAC for 48 h. JC-1 dye at a final concentration of 5 μg/ml was added to the cells 30 min before the termination of the incubation period at 37°C. Cells were harvested by trypsinization, resuspended in cold PBS, subjected to flow cytometric analysis by FACSCalibur and data were analyzed by CellQuest software (BD Biosciences, San Jose, CA).

### HPLC-MS/MS Analysis

DHA or UDC-DHA was incubated in low-glucose DMEM supplemented with 10% FBS, 2 mM L-glutamine, and antibiotics at a concentration of 20 or 2 μM respectively and an aliquot was subjected to ethyl acetate extraction and HPLC-MS/MS analysis at 0, 3, 6 or 24 h. Briefly, a 500 μl aliquot of culture medium was extracted with an equal volume of ethyl acetate. After centrifugation at 18,000 rpm for 5 min, the organic phase containing the compound was evaporated and the residue was solubilized in 500 μl mobile phase and analyzed on HPLC Dyonex Ultimate 3000 and mass spectrometer TSQ Quantum Access Max. The chromatographic separation was performed on a reverse phase Zorbax C8 column 4.6 × 150 mm, 5 μm, at flow rate of 0.5 ml/min, linear gradient H_2_O (HCOOH 1%)/acetonitrile from 20:80 to 5:95. MS/MS (ESI+) parameters: DHA precursor ion 267 [M-18], product ion 203, cone voltage 12 eV; UDC-DHA precursor ion 681 [M+23], product ion 261, cone voltage 30 eV. Limits of detection for α- and β-isomers of DHA were 0.4 and 0.9 μM respectively. Limit of detection for DHA-UDC was 0.05 nM.

### Data Analysis

Data are presented as mean ± standard error of the mean (SEM) of at least three independent experiments. IC_50_ and GI_50_ values were calculated by GraphPad Prism 6 software (GraphPad Software, San Diego, CA). Statistical analysis of data was evaluated by two-tailed Student’s *t*-test and *p*-values less than 0.05 were considered statistically significant.

## Results

### Determination of Anticancer Activity of UDC-DHA in HepG2 Cells

In a previous study, we evaluated the anticancer activity of DHA and a series of BA-DHA hybrids in HL-60 and HepG2 cells using the MTT assay, and UDC-DHA, one of the most potent BA-DHA hybrids, was 10–12 times more active than DHA in both cell lines. UDC-DHA was synthesized from DHA and UDCA by a condensation reaction mediated by 1-ethyl-3-(3-dimethylaminopropyl)carbodiimide with a yield of 61%. The purity was evaluated by ^1^H-NMR, ^13^C-NMR, MS-ESI and elemental analysis (Marchesi et al., 2019). The ^1^H-NMR and ^13^C-NMR spectra are shown in Supplementary Figures 1 and 2.

**FIGURE 2:**
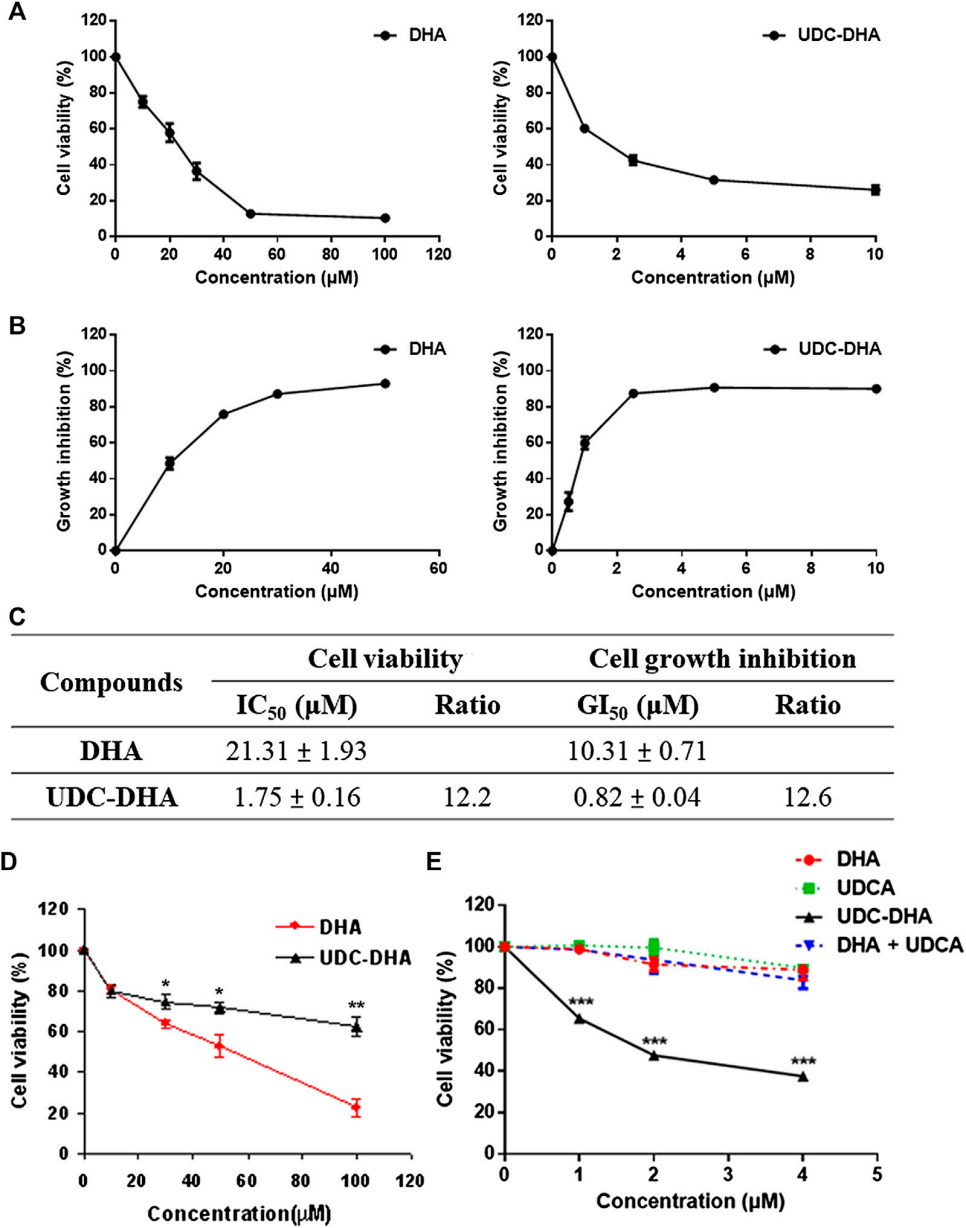
The growth inhibitory effect of DHA and UDC-DHA in HepG2 cells as well as in normal human fibroblasts. **(A)** Dose-response curves determined by the MTT assay in HepG2 cells. **(B)** Dose-response curves determined by the SRB assay in HepG2 cells. **(C)** IC_50_ or GI_50_ and the ratio of IC_50_ or GI_50_ (DHA/UDC-DHA) obtained from MTT and SRB assays. HepG2 cells were treated with indicated concentrations of DHA or UDC-DHA for 72 h, followed by the MTT or SRB assay. Data are presented as mean ± SEM of at least three independent experiments. **(D)** The dose-response curves of DHA and UDC-DHA in normal human fibroblasts. Normal human dermal fibroblasts were treated with indicated concentrations of DHA or UDC-DHA for 72 h, followed by the MTT assay. Data are presented as mean ± SEM of at least three independent experiments. Statistical significance (DHA vs. UDC-DHA) was evaluated by two-tailed Student’s *t*-test. *, *p* < 0.05; **, *p* < 0.01. **(E)** The dose-response curves of DHA, UDCA, UDC-DHA, and the combination of DHA and UDCA in HepG2 cells. HepG2 cells were treated with indicated concentrations of DHA or UDCA alone or in combination, or treated with UDC-DHA for 72 h, followed by the MTT assay. Data are presented as mean ± SEM of at least three independent experiments. Statistical significance (UDC-DHA vs. DHA, UDCA or DHA + UDCA) was evaluated by two-tailed Student’s *t*-test. ***, *p* < 0.001.

The cell viability curves over a concentration range of DHA (0–100 μM) and UDC-DHA (0–10 μM) after 72 h of treatment in HepG2 cells obtained by the MTT assay are shown in Figure 2A. The IC_50_ values of DHA and UDC-DHA were 21.31 ± 1.93 and 1.75 ± 0.16 μM respectively as reported previously (Marchesi et al., 2019). We also evaluated the growth inhibitory effect using the SRB assay, and the growth inhibition curves of DHA and UDC-DHA are illustrated in Figure 2B. The GI_50_ values were 10.31 ± 0.71 and 0.82 ± 0.04 μM, respectively. The IC_50_ and GI_50_ are summarized in Figure 2C, and GI_50_ values calculated from the SRB assay were lower than IC_50_ values obtained from the MTT assay. The IC_50_ and GI_50_ ratios between DHA and UDC-DHA remained similar with 12.2 and 12.6 respectively from the MTT and SRB assays (Figure 2C). Thus, both MTT and SRB assays confirmed that UDC-DHA was much more potent than DHA in HepG2 cells. Importantly, UDC-DHA was much less toxic to normal human fibroblasts than DHA (Figure 2D). The IC_50_ of DHA was 45.96 ± 5.11 μM, while the IC_50_ of UDC-DHA was greater than 100 μM in normal human fibroblasts. Thus, UDC-DHA showed a much better selectivity toward HepG2 cells with an IC_50_ ratio (normal cells vs. cancer cells) greater than 50 compared to DHA with a ratio of ∼2.

### Evaluation of Anticancer Activity of BA-DHA Hybrids in Huh-7 Cells

Data from HepG2 cells were very promising. We next evaluated the anticancer effect of BA-DHA hybrids in another HCC cell line Huh-7 and the results are shown in Table 1. Huh-7 cells were less sensitive to DHA with IC_50_ of 39.96 ± 1.31 µM compared to 21.3 ± 1.93 µM in HepG2 cells. However, the most potent hybrid UDC-DHA was almost as potent in Huh-7 (IC_50_: 2.16 ± 0.39 µM, DHA/hybrid IC_50_ ratio: 18.53) as in HepG2 cells (IC^50^ of 1.75 ± 0.16 µM, DHA/hybrid IC50 ratio: 12.2). The building blocks were also tested in Huh-7 cells but the cytotoxicity was very low with IC_50_ values all greater than 100 µM (Supplementary Table 1). It has been reported that artemisinin and its derivatives inhibit HCC cell growth regardless of the p53 status (Hou et al., 2008). However, it has also been reported that p53 facilitates apoptosis induced by DHA in HCC cells (Zhang et al., 2012). Interestingly, HepG2 cells have wild-type p53, while Huh-7 cells express a mutant p53 (p53^Y220C^), suggesting that UDC-DHA may have growth inhibitory effect in hepatocellular carcinoma cells independent of the p53 status.

**TABLE 1:** IC_50_ values of DHA and BA-DHA hybrids in Huh-7 cells (72 h treatment).

Compound	IC_50_ (µM)^a^	DHA/Hybrid^b^
DHA	39.96 ± 1.31	—
CDC-DHA	14.41 ± 2.18	2.77
N_3_CDC-DHA	50.30 ± 2.45	0.78
DHA-CDC-DHA	>100	<0.40
**UDC-DHA**	**2.16 ± 0.39**	**18.53**
N_3_UDC-DHA	4.13 ± 0.84	9.68
HDC-DHA	6.03 ± 0.68	6.62
N_3_HDC-DHA	15.92 ± 1.28	2.72
LC-DHA	5.97 ± 0.74	6.69
N_3_LC-DHA	5.08 ± 0.44	8.18

a Data are presented as mean ± SEM of at least three independent experiments.

b The DHA/Hybrid value was calculated as the ratio of the IC_50_ of DHA and the hybrid.

The most potent hybrid UDC-DHA is highlighted in bold.

#### Covalent linkage between UDCA and DHA is important for enhancing the anticancer activity of DHA

The growth inhibitory effect of DHA, UDCA, UDC-DHA and the combination of DHA and UDCA at a 1:1 molar ratio in the concentration range of 0–4 μM was compared in HepG2 cells. As shown in Figure 2E, DHA, UDCA, and the combination of DHA and UDCA barely affected cell growth in the low concentration range tested, whereas UDC-DHA was significantly more potent, suppressing more than 60% of cell growth at 4 μM. These results demonstrated that the enhanced anticancer activity of UDC-DHA was due to a covalent linkage, but not a synergistic effect between UDCA and DHA.

### Effects of UDC-DHA on Cell Cycle Progression and Apoptosis

**FIGURE 3:**
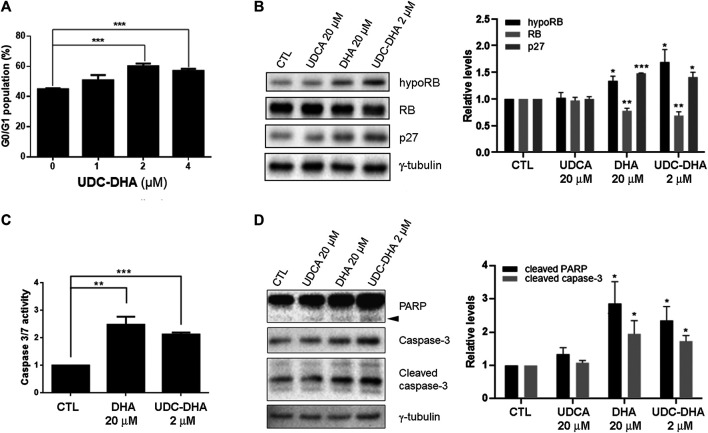
Effects of UDC-DHA on cell cycle progression and apoptosis in HepG2 cells. **(A)** Treatment with UDC-DHA for 24 h increased the G0/G1 population in a dose-dependent manner. HepG2 cells were treated with UDC-DHA for 24 h followed by PI staining and flow cytometric analysis. **(B)** DHA and UDC-DHA increased the levels of G0/G1 protein markers hypophosphorylated RB (hypoRB) and p27 in HepG2 cells. Cells were treated with indicated concentrations of DHA, UDCA and UDC-DHA for 24 h and harvested for Western blot analysis. **(C)** DHA and UDC-DHA induced caspase 3/7 activity in HepG2 cells. Cells were treated with indicated concentrations of DHA and UDC-DHA for 72 h and subjected to the Caspase-Glo 3/7 assay. **(D)** DHA and UDC-DHA increased the cleavage of PARP and casapse-3 in HepG2 cells. Cells were treated with indicated concentrations of DHA, UDCA and UDC-DHA for 72 h and harvested for Western blot analysis. Cleaved PARP is marked by an arrowhead. Relative protein levels were quantified by Image Lab software and γ-tubulin was used as a loading control. Data are presented as mean ± SEM of at least three independent experiments. Statistical significance was evaluated by two-tailed Student’s *t*-test compared to the vehicle control (CTL). *, *p* < 0.05; **, *p* < 0.01; ***, *p* < 0.001.

It has been reported that DHA affects cell cycle progression and induces G1 arrest in HepG2 cells (Hou et al., 2008). To evaluate the effect of UDC-DHA on the cell cycle, HepG2 cells treated with 0–4 μM of UDC-DHA for 24 h were subjected to PI staining and flow cytometric analysis. As shown in Figure 3A, UDC-DHA increased the G0/G1 population in a dose-dependent manner. Western blot analysis also revealed that 2 μM of UDC-DHA induced hypophosphorylated RB and p27, and downregulated the total RB protein. Similar effects were observed in HepG2 cells treated with 20 μM of DHA (approximately equivalent to 2 μM of UDC-DHA in potency) but not 20 μM of UDCA for 24 h (Figure 3B). Thus, both DHA and UDC-DHA induced G0/G1 cell cycle arrest in HepG2 cells. To determine whether DHA or UDC-DHA induced apoptosis, HepG2 cells were first treated with 20 μM of DHA or 2 μM of UDC-DHA for 72 h and then subjected to the Caspase-Glo 3/7 assay. Results shown in Figure 3C indicated that both 20 μM of DHA and 2 μM of UDC-DHA significantly induced apoptosis compared to the DMSO vehicle control in HepG2 cells (2.48-fold and 2.13-fold respectively relative to the vehicle control). The results were further confirmed by Western blot analysis. HepG2 cells were treated with 2 μM of UDC-DHA, 20 μM of DHA or 20 μM of UDCA for 72 h followed by Western blot analysis of apoptotic markers including cleaved PARP and caspase-3. As illustrated in Figure 3D, both 20 μM DHA and 2 μM UDC-DHA significantly increased the levels of cleaved PARP (2.86-fold and 2.35-fold) and cleaved caspase-3 (1.95-fold and 1.73-fold) compared to the vehicle control, while 20 μM UDCA did not have any obvious effect. UDC-DHA also increased G0/G1 population and induced subG1 cells in Huh-7 cells (Supplementary Figure 3). Altogether, these results indicated that UDC-DHA may induce G0/G1 arrest and subsequently apoptosis in HCC cells.

### DHA and UDC-DHA Induce ROS Generation in HepG2 Cells but With Different Magnitude and Timing

**FIGURE 4:**
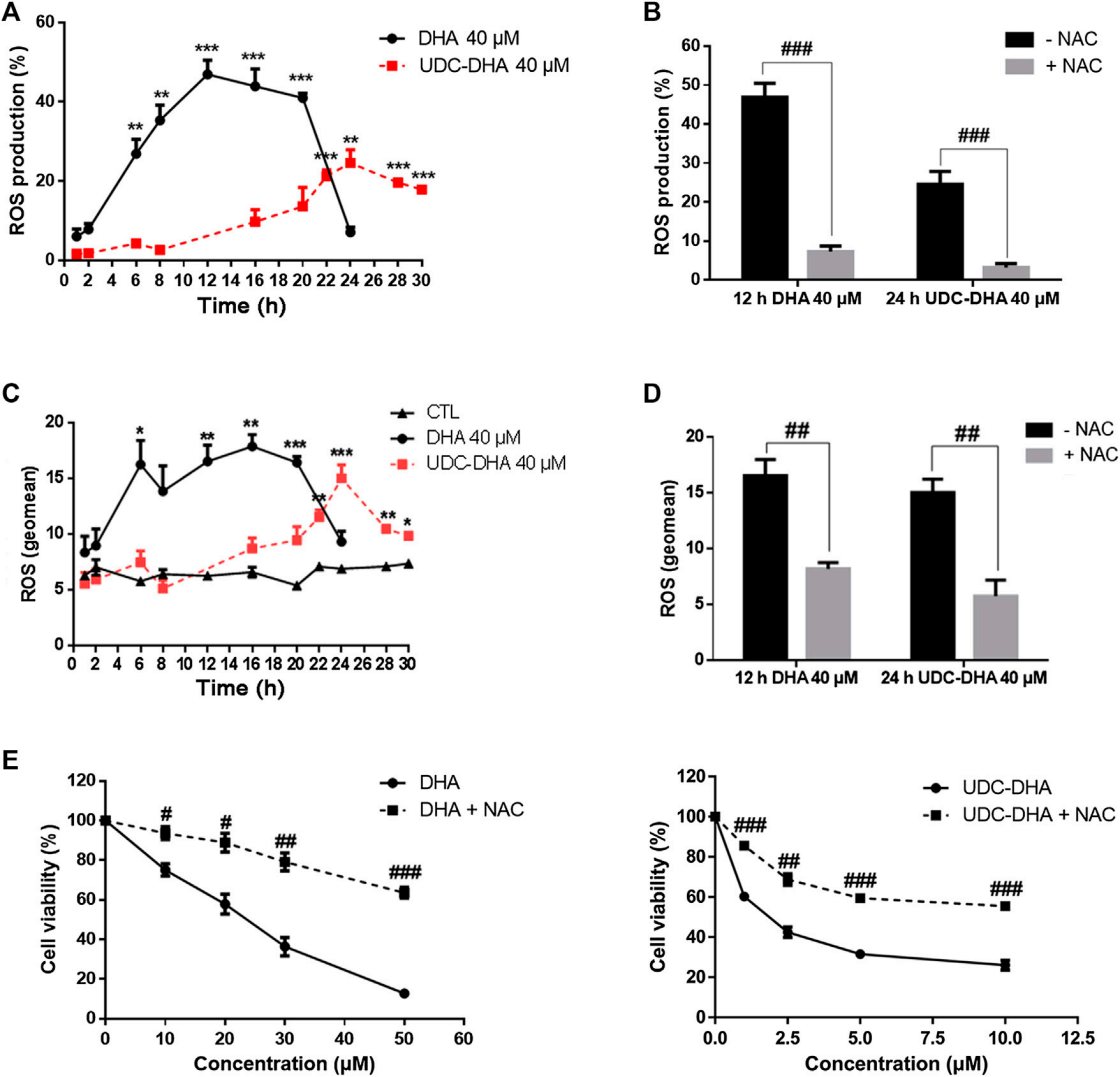
Effects of DHA and UDC-DHA on ROS generation in HepG2 cells. DHA and UDC-DHA induced ROS over time in HepG2 cells **(A,C)**, and NAC reversed ROS generation induced by DHA and UDC-DHA **(B,D)**. HepG2 cells were treated with indicated concentrations of DHA or UDC-DHA for various time periods with or without 2 mM of NAC. DCFH-DA (10 μM) was added to the cells 30 min before termination of the treatment. Cells were then harvested for flow cytometric analysis of DCF fluorescence. The percentages of cells with ROS production (**A,B**) or the geomean of ROS (**C,D**) were analyzed by FlowJo software. (**E**) NAC reversed the growth inhibitory effect of DHA and UDC-DHA. HepG2 cells were treated with various concentrations of DHA or UDC-DHA in the absence or presence of 2 mM of NAC for 72 h, followed by the MTT assay to measure cell viability. Data are presented as mean ± SEM of at least three independent experiments. *, *p* < 0.05; **, *p* < 0.01; ***, *p* < 0.001 vs. the control group. #, *p* < 0.05; ##, *p* < 0.01; ###, *p* < 0.001 vs. respective non-NAC treatment group.

It is well accepted that DHA can generate ROS through cleavage of the endoperoxide bridge (Efferth, 2017; Wong et al., 2017). We reported previously that UDC-DHA induced significantly more ROS than DHA in HepG2 cells after 24 h of treatment (Marchesi et al., 2019). Here, we further determined ROS induction by DHA or UDC-DHA over time and found distinct induction patterns in HepG2 cells. As shown in Figure 4A, by setting the ROS production in the vehicle treated control cells as 5% (i.e. 5% ROS positive cells), 40 μM of DHA dramatically elevated ROS production which peaked at 12 h (46.93 ± 3.57%), remained high until 20 h and then declined rapidly to less than 10% at 24 h, while 40 μM of UDC-DHA gradually increased ROS production which peaked at 24 h (24.56 ± 3.31%) and persisted until 30 h with a mild decline. The induction of ROS by DHA at 12 h and UDC-DHA at 24 h was markedly reversed in the presence of an ROS scavenger NAC (Figure 4B). An alternative way to analyze ROS production was to calculate the geomean and the geomean of ROS induced by 40 μM of DHA was higher and reached the maximum (16.58 ± 1.44 at 12 h and 17.93 ± 1.04 at 16 h) earlier than that induced by 40 μM of UDC-DHA (15.06 ± 1.19 at 24 h) (Figure 4C). The geomean of ROS induced by DHA at 12 h or by UDC-DHA at 24 h was also significantly reduced in the presence of NAC (Figure 4D). In addition, NAC also significantly reversed the growth inhibitory effect of DHA and UDC-DHA (Figure 4E). Taken together, these results suggested that although the induction of ROS by DHA and UDC-DHA varied in magnitude and timing, ROS generation may play an important role in the anticancer activity of DHA and UDC-DHA in HepG2 cells. In Huh-7 cells, 40 μM of DHA also elevated ROS production over time which peaked at 6 h and then declined at 24 h; however, 40 μM of UDC-DHA did not induce ROS generation at all (Supplementary Figure 4).

### UDC‐DHA Causes More Severe MMP Loss Than DHA in Hepatocellular Carcinoma Cells

**FIGURE 5:**
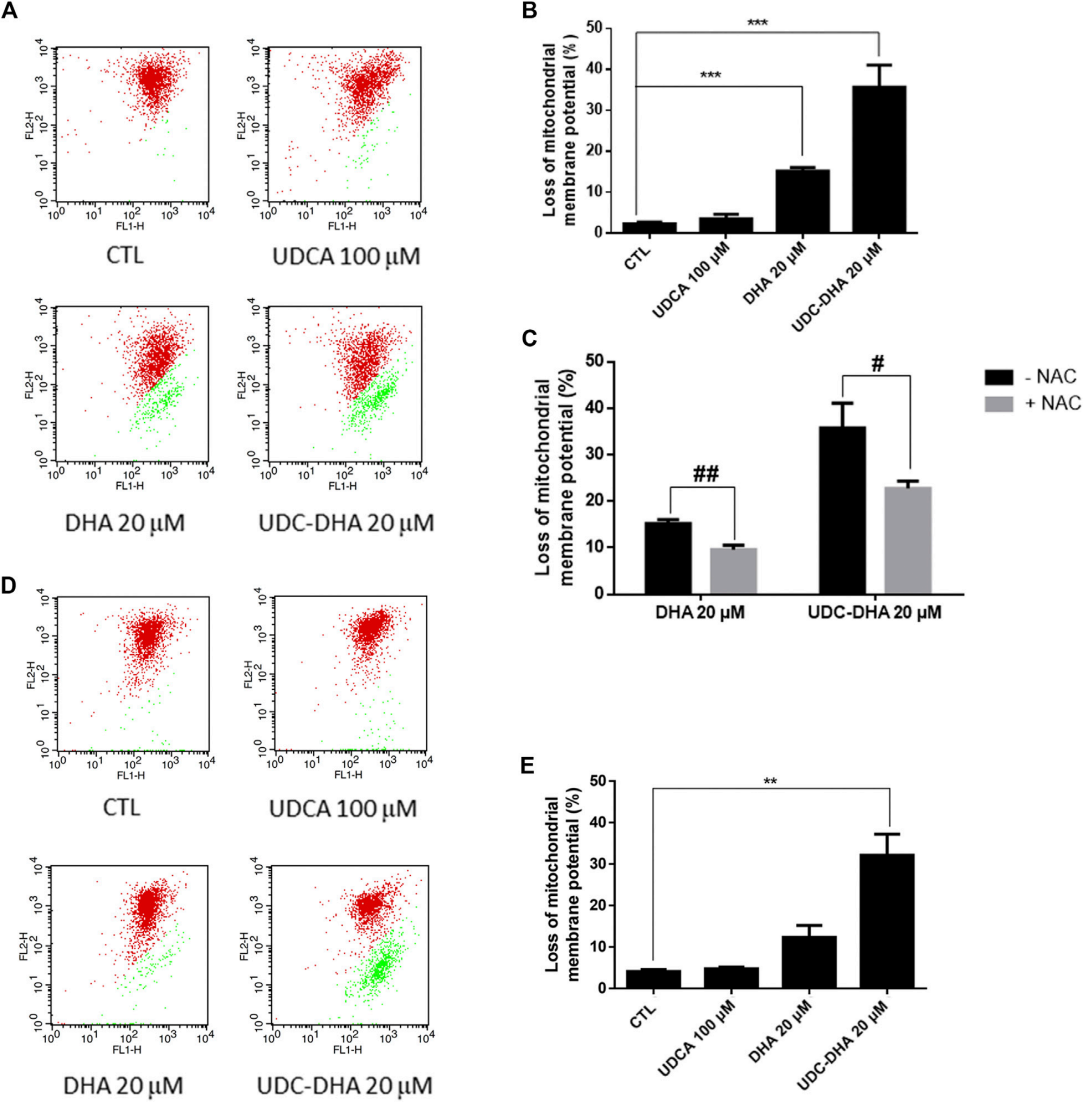
DHA and UDC-DHA induce MMP loss in HCC cells. **(A)** Dot plots of MMP loss induced by DHA, UDCA and UDC-DHA in HepG2 cells. **(B)** Quantitative data of MMP loss induced by DHA, UDCA and UDC-DHA in HepG2 cells. **(C)** NAC cotreatment reversed the depolarization of MMP induced by DHA and UDC-DHA in HepG2 cells. **(D)** Dot plots of MMP loss induced by DHA, UDCA and UDC-DHA in Huh-7 cells. **(E)** Quantitative data of MMP loss induced by DHA, UDCA and UDC-DHA in Huh-7 cells. HCC cells were treated with indicated concentrations of DHA, UDCA and UDC-DHA for 48 h with or without 2 mM NAC. JC-1 dye (5 μg/ml) was added to the cells 30 min before termination of the treatment period. Cells were harvested for flow cytometric analysis of JC-1 fluorescence. The percentage of cells with depolarization of MMP (labeled in green) was analyzed by CellQuest software. Data are presented as mean ± SEM of at least three independent experiments. **, *p* < 0.01; ***, *p* < 0.001 vs. the control group. #, *p* < 0.05; ##, *p* < 0.01 vs. respective non-NAC treatment group.

Since ROS has been reported to be responsible for the loss of MMP induced by DHA in HepG2 cells (Qin et al., 2015), we next determined whether DHA, UDCA or UDC-DHA treatment led to the depolarization of MMP in HepG2 cells by JC-1 staining and flow cytometric analysis. JC-1 is a membrane-permeable cationic dye which is accumulated in mitochondria of living cells in a membrane potential-dependent manner and forms aggregates with red fluorescence. When the mitochondrion is impaired and MMP is lost, JC-1 monomers become dominant and display green fluorescence. A set of dot plots is shown in Figure 5A and quantitative data are illustrated in Figure 5B. Loss of MMP was significantly induced by 20 μM DHA (15.17 ± 0.88%) and 20 μM UDC-DHA (35.73 ± 5.38%) but not 100 μM UDCA. Interestingly, 20 μM UDC-DHA caused more severe MMP loss than 20 μM DHA. Moreover, as shown in Figure 5C, cotreatment with NAC reduced the loss of MMP induced by DHA and UDC-DHA in HepG2 cells, indicating that ROS may act as an upstream factor of MMP loss. In spite of the lack of ROS production, more severe MMP loss was also induced by UDC-DHA in Huh-7 cells (Figures 5D,E).

### UDC_DHA and is More Stable Than DHA That may Account for its Enhanced Anticancer Activity

**FIGURE 6:**
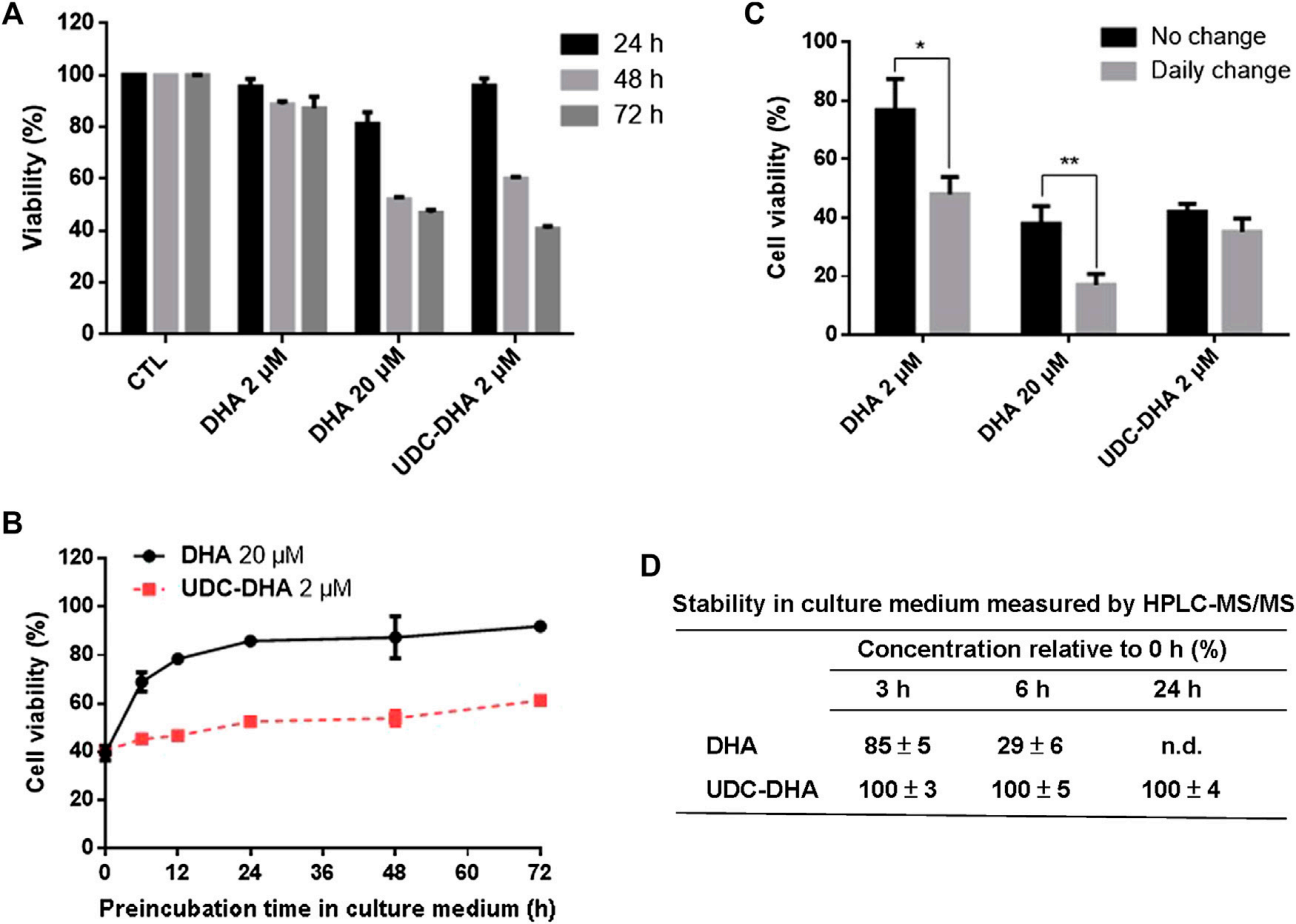
UDC-DHA is more stable than DHA. **(A)** Time course of the effect of DHA and UDC-DHA on cell viability revealed that UDC-DHA acted gradually and more persistently than DHA. **(B)** Pre-incubation of DHA but not UDC-DHA in culture medium greatly reduced growth inhibitory effect. DHA and UDC-DHA were incubated in culture medium at 37°C for various time periods and then added to HepG2 cells for 72 h, followed by the MTT assay. Results were obtained from at least three independent experiments and data are presented as mean ± SEM. **(C)** Daily change of culture medium containing DHA but not UDC-DHA significantly increased the growth inhibitory effect. HepG2 cells were treated with indicated concentrations of DHA or UDC-DHA for 72 h with or without daily change of drug containing medium and then subjected to the MTT assay. Results are presented as mean ± SEM of three independent experiments. *, *p* < 0.05; **, *p* < 0.01. **(D)** Stability of DHA and UDC-DHA in cell culture medium measured by HPLC-MS/MS. DHA (20 μM) or UDC-DHA (2 μM) was incubated in cell culture medium and 500 μl aliquots were extracted with ethyl acetate for HPLC-MS/MS analysis. The concentrations of DHA and UDC-DHA at 3, 6 or 24 h were calculated by comparison with that of the 0 h. The data are presented as mean ± SEM of three experiments. n.d., not detected.

DHA has a short half-life. We speculated that conjugation of DHA with bile acids may stabilize DHA, therefore enhance its anticancer activity. To test this hypothesis, we first conducted time course experiments to compare the viability of HepG2 cells following the treatment with 2 μM DHA, 20 μM DHA and 2 μM UDC-DHA for 24, 48 or 72 h. As illustrated in Figure 6A, 2 μM DHA or UDC-DHA barely exhibited any significant growth inhibitory effect at 24 h, whereas 20 μM DHA significantly inhibited cell viability down to 81.3 ± 4.37% (*p* = 0.013 vs. C) at 24 h. At 48 h, 20 μM DHA continued to decrease cell viability down to 52.1 ± 0.997% and 2 μM UDC-DHA started to show clear growth inhibitory effect with 60.0 ± 0.84% cell viability in contrast to 2 μM DHA with 88.8 ± 1.25% cell viability. Interestingly, at 72 h, the growth suppression effect of 2 μM UDC-DHA had surpassed that of 20 μM DHA (cell viability: 40.8 ± 0.97% vs. 46.9 ± 1.15%, *p* = 0.015), whereas 2 μM DHA only led to 87.3 ± 4.46% cell viability at this time (Figure 6A). These results indicated that UDC-DHA acted gradually and more persistently than DHA which could be due to increased stability.

To verify this possibility, 20 μM DHA and 2 μM UDC-DHA were pre-incubated in culture medium for various time periods at 37°C and then added to HepG2 cells for 72 h, followed by the MTT assay. As shown in Figure 6B, a time-dependent loss of DHA activity was observed when it was pre-incubated in culture medium for 6–72 h. The growth inhibitory effect of DHA was reduced by half after pre-incubation for 6 h in culture medium (39.59 ± 3.02% cell viability without pre-incubation vs. 68.88 ± 3.97% cell viability after 6 h of pre-incubation) and the activity was almost abolished after 48 h of pre-incubation in culture medium (87.35 ± 8.75% viability). On the contrary, the activity of UDC-DHA was more stable than DHA (40.57 ± 0.62% cell viability without pre-incubation vs. 53.92 ± 3.10% and 61.28 ± 0.66% cell viability after 48 and 72 h of pre-incubation respectively).

To further verify the stability of DHA and UDC-DHA, HepG2 cells were treated with the indicated conditions of DHA and UDC-DHA with or without daily change of drug-containing medium for 72 h and cell viability was measured by the MTT assay. Figure 6C showed that the cell viability of both 2 and 20 μM DHA treated cells with daily change was markedly reduced than the respective no-change group. In contrast, there was no significant difference between cells treated with 2 μM UDC-DHA either with or without daily change.

The chemical stability of DHA and UDC-DHA in cell culture medium was also determined by HPLC-MS/MS analysis. Cell culture medium containing 20 μM DHA or 2 μM UDC-DHA was subjected to ethyl acetate extraction and HPLC-MS/MS analysis after 0, 3, 6 or 24 h of incubation and the relative concentration (% of the concentration at 0 h) was calculated. As illustrated in Figure 6D, the concentration of DHA was decreased by 15% after 3 h (85 ± 5%), by 71% after 6 h (29 ± 6%) and was undetectable after 24 h of incubation. In contrast, the concentrations of UDC-DHA remained unchanged at all the time points tested. Examples of HPLC-MS/MS chromatograms are shown in Supplementary Figure 5 (DHA) and Figure 6 (UDC-DHA). Altogether, these results indicated that DHA was unstable and its cytotoxic effect diminished dramatically over time; whereas, UDC-DHA was more stable in culture medium and probably inside the cell as well that may in part explain why UDC-DHA was a more potent anticancer agent than DHA.

### The Enhanced Activity of UDC_DHA may not Be Attributed to Cellular Targeting via Bile Acid Transporters

**FIGURE 7:**
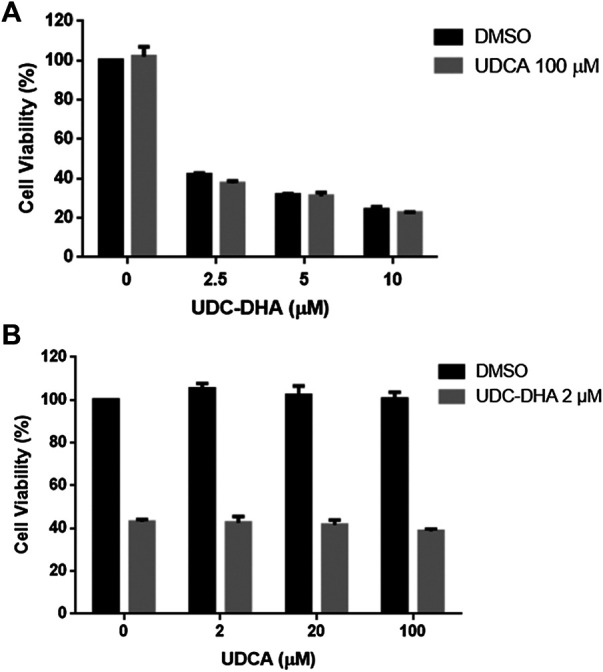
An excess amount of UDCA does not affect growth inhibitory effect of UDC-DHA. **(A)** HepG2 cells were treated with indicated concentrations of UDC-DHA in the absence or presence of 100 μM UDCA for 72 h, followed by the MTT assay. **(B)** HepG2 cells were treated with increasing amount of UDCA in the absence or presence of 2 μM of UDC-DHA. Data are presented as mean ± SEM of at least three independent experiments.

UDC-DHA was much more potent than DHA. Since HepG2 cells express bile acid transporters (Obaidat et al., 2012), the bile acid moiety could target UDC-DHA to bile acid transporters on HepG2 cells, thereby enhancing drug uptake by the cells. If so, UDCA might compete with UDC-DHA and reverse the growth inhibitory effect of UDC-DHA. To test this hypothesis, HepG2 cells were treated with 0–10 μM of UDC-DHA in the absence or presence of 100 μM UDCA (Figure 7A) or with an increasing amount of UDCA in the absence or presence of 2 μM of UDC-DHA (Figure 7B) for 72 h, and then subjected to the MTT assay. The results revealed that an excess amount of UDCA was unable to reverse the growth inhibitory effect of UDC-DHA, suggesting that the enhanced activity of UDC-DHA in HepG2 cells may not be due to cellular targeting via bile acid transporters.

## Discussion

The anticancer activity of a series of BA-DHA hybrids were tested in a HCC cell line HepG2 using the MTT assay in a previous study. Among them, UDC-DHA was one of the most potent hybrids and it was ∼12 times more active than DHA (Marchesi et al., 2019). Here we also evaluated the activity of BA-DHA hybrids in another HCC cell line Huh-7 and UDC-DHA was the most potent BA-DHA hybrid with an average IC_50_ value of 2.16 μM and a DHA/hybrid IC_50_ ratio of 18.53. HepG2 cells have wild-type p53, while Huh-7 cells express a mutant p53 (p53^Y220C^). Although less sensitive to DHA, Huh-7 cells were almost as sensitive to UDC-DHA compared to HepG2 cells, suggesting that the anticancer effect of UDC-DHA may be less affected by the p53 status in HCC cells. Therefore, the mechanism of the enhanced anticancer activity of UDC-DHA was investigated in this study. It has been reported that UDCA in combination with sorafenib, the first-line drug for patients with advanced HCC, has shown a synergistic effect (Lee et al., 2018). However, more than 750 μM of UDCA was required to achieve this effect. The cytotoxicity toward HepG2 cells was compared between the UDC-DHA treatment group and the combination of UDCA and DHA at a 1:1 molar ratio. Our results showed that UDC-DHA was more potent than the combination group and no synergistic effect between DHA and UDCA was observed in the low concentration range used in this study (Figure 2E). Thus, as reported previously in HL-60 cells (Marchesi et al., 2019), a covalent linkage rather than a synergistic effect between UDCA and DHA is responsible for the increased anticancer activity of UDC-DHA in HepG2 cells.

Previous studies have shown that DHA can induce cell cycle arrest and apoptosis in HCC cells (Hou et al., 2008; Zhang et al., 2012; Qin et al., 2015). However, DHA has been reported to induce G0/G1 arrest (Hou et al., 2008) or G2/M arrest (Zhang et al., 2012) in HepG2 cells. Our cell cycle analysis revealed that UDC-DHA in the low concentration range (2 and 4 μM) significantly increased G0/G1 populations in both HepG2 and Huh-7 cells. Western blot analysis also revealed that both DHA and UDC-DHA induced hypophosphorylation of RB and downregulation of total RB protein, as well as increased p27 protein levels, confirming their effect on G0/G1 arrest in HepG2 cells (Figures 3A,B). UDC-DHA at 2 μM also markedly induced apoptosis comparable to the effect of 20 μM DHA based on the Caspase-3/7 activity assay as well as Western blot analysis of cleaved PARP and caspase-3 in HepG2 cells (Figures 3C,D). UDC-DHA at 2 μM also significantly induced subG1 population, albeit less effective than 40 μM DHA in Huh-7 cells (Supplementary Figure 3) possibly due to p53 mutation since p53 may facilitate apoptosis induction (Zhang et al., 2012). Collectively, these data suggested that UDC-DHA was capable of inducing G0/G1 arrest and apoptosis at a much lower concentration than DHA in HCC cells.

The mechanism of the antimalarial action of DHA remains a topic of debate, but the widely accepted theory is that DHA generates ROS which then alkylate and oxidize proteins, resulting in the death of the parasite (Krungkrai and Krungkrai, 2016). Moreover, DHA has been reported to elevate ROS levels and induce apoptosis in several cancer cell lines (Wang et al., 2012; Qin et al., 2015). We have shown previously that UDC-DHA induced significantly more ROS than DHA in HepG2 cells treated with 40 μM of each for 24 h, seemingly correlated with their anticancer activities (Marchesi et al., 2019). In this study, however, we observed that DHA and UDC-DHA induced different ROS generation patterns over time, which may be related to the stability of both compounds. As illustrated in Figure 4, ROS production caused by DHA was more dramatic, peaked early at 12–16 h and declined at 24 h; whereas UDC-DHA induced relatively less ROS which peaked at 24 h. According to the stability assay, DHA was unstable in culture medium and possibly also in cells (Figure 6). Thus, DHA might induce ROS quickly and markedly, and then the effect was reduced dramatically. In contrast, since UDC-DHA was more stable, its ROS production persisted over a longer time period. It has been reported that a DHA-cinnamic acid ester derivative compound **17** displayed an IC_50_ value of 0.2 μM in A549 cells but 30 μM of the compound was required for clear ROS induction (Xu et al., 2016). Similarly, 40 μM UDC-DHA was required to induce detectable ROS production in HepG2 cells. A possible explanation is that the DHA moiety with an endoperoxide bridge in UDC-DHA is responsible for the ROS induction and ROS induced by low concentration of UDC-DHA may be below the detection limit. The area under the ROS time course of UDC-DHA was much less than that of DHA, suggesting that the ROS elevation induced by UDC-DHA may not be the main reason for causing HCC cell death. Nevertheless, a ROS scavenger NAC reversed the UDC-DHA-induced cytotoxicity. Recently, a ROS threshold theory has been proposed (Galadari et al., 2017). Cancer cells have a high level of endogenous ROS that can be further increased by therapies to elevate ROS generation to a toxic level and induce death in cancer cells. Cancer cells die once the ROS level reaches the death threshold. Thus, although UDC-DHA could not induce higher ROS production than DHA, it may still cause cancer cell death as long as the ROS level has reached the death threshold. Detection of MMP loss through JC-1 staining and flow cytometric analysis showed that UDC-DHA induced a higher degree of depolarization of MMP than DHA at the same concentration (20 μM) in HepG2 cells. Moreover, NAC reversed the depolarization of MMP induced by both DHA and UDC-DHA, indicating that ROS may be responsible for the loss of MMP in HepG2 cells. Surprisingly, only DHA but not UDC-DHA induced ROS in Huh-7 cells (Supplementary Figure 4). However, UDC-DHA caused more severe MMP loss than DHA in Huh-7 cells as in HepG2 cells (Figure 5), suggesting that MMP loss may be more related to its activity in Huh-7 cells.

Interestingly, UDC-DHA exhibited a much better selectivity toward HepG2 cells with an IC_50_ ratio of normal and cancer cells greater than 50 while DHA had a ratio of ∼2, indicating that the conjugation with UDCA also greatly increased the selectivity of DHA. UDCA is a remarkable molecule exhibiting both anti- and pro-apoptotic properties toward different cell types depending on the conditions (Goossens and Bailly, 2019). We also reported on the selective cytotoxicity of several UDCA-based conjugates (Navacchia et al., 2016; Navacchia et al., 2017). In general, normal cells have a slower growth rate that may in part account for the lower sensitivity to anticancer drugs. Since ROS induction and MMP loss were associated with the activity of UDC-DHA in HepG2 cells, we measured ROS production and depolarization of MMP in normal human fibroblast cells after UDC-DHA treatment and found that both ROS induction and MMP loss in NHDF cells were less than in HepG2 cells (Supplementary Figure 7). This may also partly account for the selectivity. However, the reason why UDC-DHA has a better selectivity than DHA is still unclear and requires further investigation.

Previous studies have demonstrated that a series of BA derivatives conjugated with cisplatin may target liver tumors through the uptake by transporters, including organic cation transporter, organic anion transporting polypeptide (OATP) or Na^+^-taurocholate cotransporting polypeptide (Dominguez et al., 2001; Briz et al., 2002). Additionally, the K_m_ values for bile acid transporters, such as OATPs reported to be overexpressed in cancer cells (Buxhofer-Ausch et al., 2013), were 14–60 μM (St-Pierre et al., 2001). We hypothesized that if UDC-DHA could be targeted to HepG2 cells via bile acid transporters, its anticancer activity might be subdued by the presence of UDCA. However, cotreatment of 100 μM UDCA with 0–10 μM UDC-DHA or an increasing amount of UDCA with 2 μM UDC-DHA had no significant effect on growth inhibition caused by UDC-DHA in HepG2 cells. Furthermore, the concentrations of UDC-DHA used in this study were lower than the K_m_ values for bile acid transporters. Thus, it is unlikely that the enhanced activity of UDC-DHA is due to targeting via bile acid transporters in our *in vitro* assay system. Whether targeting via bile acid transporters influence *in vivo* activity of UDC-DHA remains to be determined.

It has been demonstrated that the antimalarial activity of DHA was almost completely abolished after 24 h-incubation in plasma (Parapini et al., 2015). Time course experiments indicated that a high concentration of DHA exerted the growth inhibitory effect faster and reached the maximum effect at 48 h, whereas a low concentration of UDC-DHA with approximately equivalent potency acted gradually and reached its maximum effect at the end of the 72 h incubation period (Figure 6A), suggesting that UDC-DHA was more stable. To verify this, DHA and UDC-DHA were pre-incubated in culture medium for various time periods before added to the cells for activity assay. The results showed that the activity of DHA dropped dramatically after pre-incubation for 6 h, while the activity of UDC-DHA was in a steady state after incubation in the culture medium (Figure 6B). Moreover, there was no significant difference in cell viability between the no-change group and the daily-change group of UDC-DHA, but daily change of DHA-containing medium greatly enhanced the growth inhibitory effect (Figure 6C). HPLC-MS/MS analysis also revealed that the chemical stability of UDC-DHA was far more superior to DHA in cell culture medium (Figure 6D). Taken together, these results indicated that UDC-DHA was more stable than DHA, which may at least in part account for the enhanced anticancer activity of UDC-DHA.

It has been proposed that the lactol ring of DHA can be opened and closed allowing the switch of the hydroxyl group between the α and β positions, that may influence the stability of the seven membered ring with the peroxide bridge (Jansen, 2010). By forming an ester linkage with DHA through the hydroxyl group, the bile acid moiety may prevent ring opening and stabilize the heterocyclic ring system of DHA. Furthermore, the main metabolic fate of DHA is the formation of α-DHA-β-glucuronide via the hydroxyl group catalyzed by UDP-glucuronosyltransferase (UGT) isoforms, UGT1A9 and UGT2B7 (Ilett et al., 2002). The bile acid moiety of the BA-DHA hybrid protects DHA from glucuronidation, thereby, may increase the intracellular stability of BA-DHA unless DHA is released from the hybrid by esterases. Thus, UDC-DHA may act as a prodrug not only to improve the chemical and metabolic stability but also to sustain the release of the active drug DHA. There is also a possibility that the intact UDC-DHA hybrid can be an active drug. Further studies are needed to clarify these issues.

## Conclusion

**FIGURE 8:**
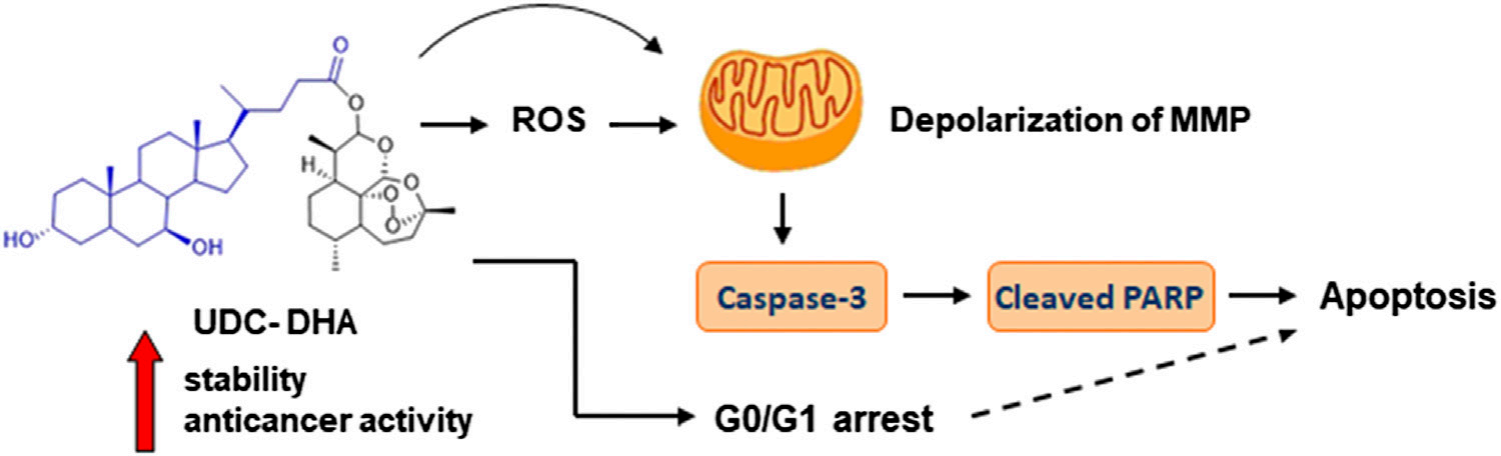
UDC-DHA induces G0/G1 arrest and apoptosis in HCC cells with markedly increased stability and anticancer activity compared to DHA.

Many studies have raised the possibility of repurposing DHA as an anticancer agent. However, its poor stability is a major concern. Here we report that a BA-DHA hybrid UDC-DHA is more active than DHA in both HepG2 and Huh-7 HCC cells. Mechanism study shows that similar to DHA, UDC-DHA induces G0/G1 arrest in both HepG2 and Huh-7 cells. UDC-DHA also elevates ROS levels in HepG2 but not in Huh-7 cells, and causes depolarization of MMP in both HepG2 and Huh-7 cells. These effects may ultimately contribute to apoptosis induction as depicted in Figure 8. Importantly, UDC-DHA is more stable than DHA that may account for the increased potency of UDC-DHA. Furthermore, UDC-DHA is less toxic to normal cells than DHA and has a much better selectivity toward HCC cells. Thus, UDC-DHA could be a better drug candidate than DHA for the treatment of HCC and deserves further investigations.

## Data Availability

The original contributions presented in the study are included in the article/Supplementary Materials, further inquiries can be directed to the corresponding authors.
