# Supplementary material for: Evaluation of the Anticancer Activity of a Bile Acid-Dihydroartemisinin Hybrid Ursodeoxycholic-Dihydroartemisinin in Hepatocellular Carcinoma Cells
Source: Front Pharmacol. 2020 Nov 10;11:599067. doi: 10.3389/fphar.2020.599067 (PMC7748086; doi:10.3389/fphar.2020.599067)
Supplement: Supplementary file 1 [file Presentation1_v1.PPTX]

## Slide 1
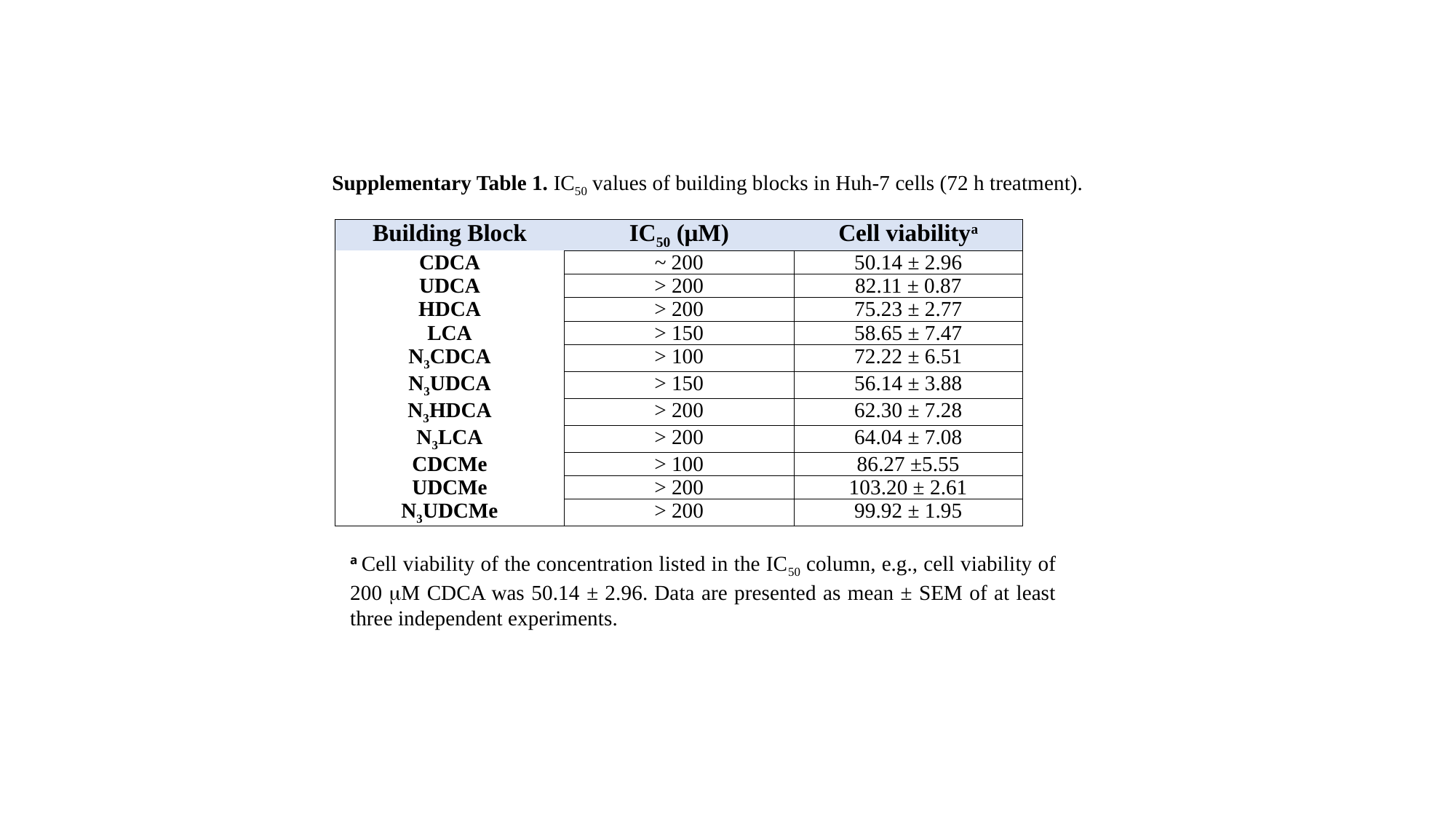

Supplementary Table 1. IC50 values of building blocks in Huh-7 cells (72 h treatment).
| Building Block | IC50 (μM) | Cell viabilitya |
| --- | --- | --- |
| CDCA | ~ 200 | 50.14 ± 2.96 |
| UDCA | > 200 | 82.11 ± 0.87 |
| HDCA | > 200 | 75.23 ± 2.77 |
| LCA | > 150 | 58.65 ± 7.47 |
| N3CDCA | > 100 | 72.22 ± 6.51 |
| N3UDCA | > 150 | 56.14 ± 3.88 |
| N3HDCA | > 200 | 62.30 ± 7.28 |
| N3LCA | > 200 | 64.04 ± 7.08 |
| CDCMe | > 100 | 86.27 ±5.55 |
| UDCMe | > 200 | 103.20 ± 2.61 |
| N3UDCMe | > 200 | 99.92 ± 1.95 |
a Cell viability of the concentration listed in the IC50 column, e.g., cell viability of 200 μM CDCA was 50.14 ± 2.96. Data are presented as mean ± SEM of at least three independent experiments.

## Slide 2
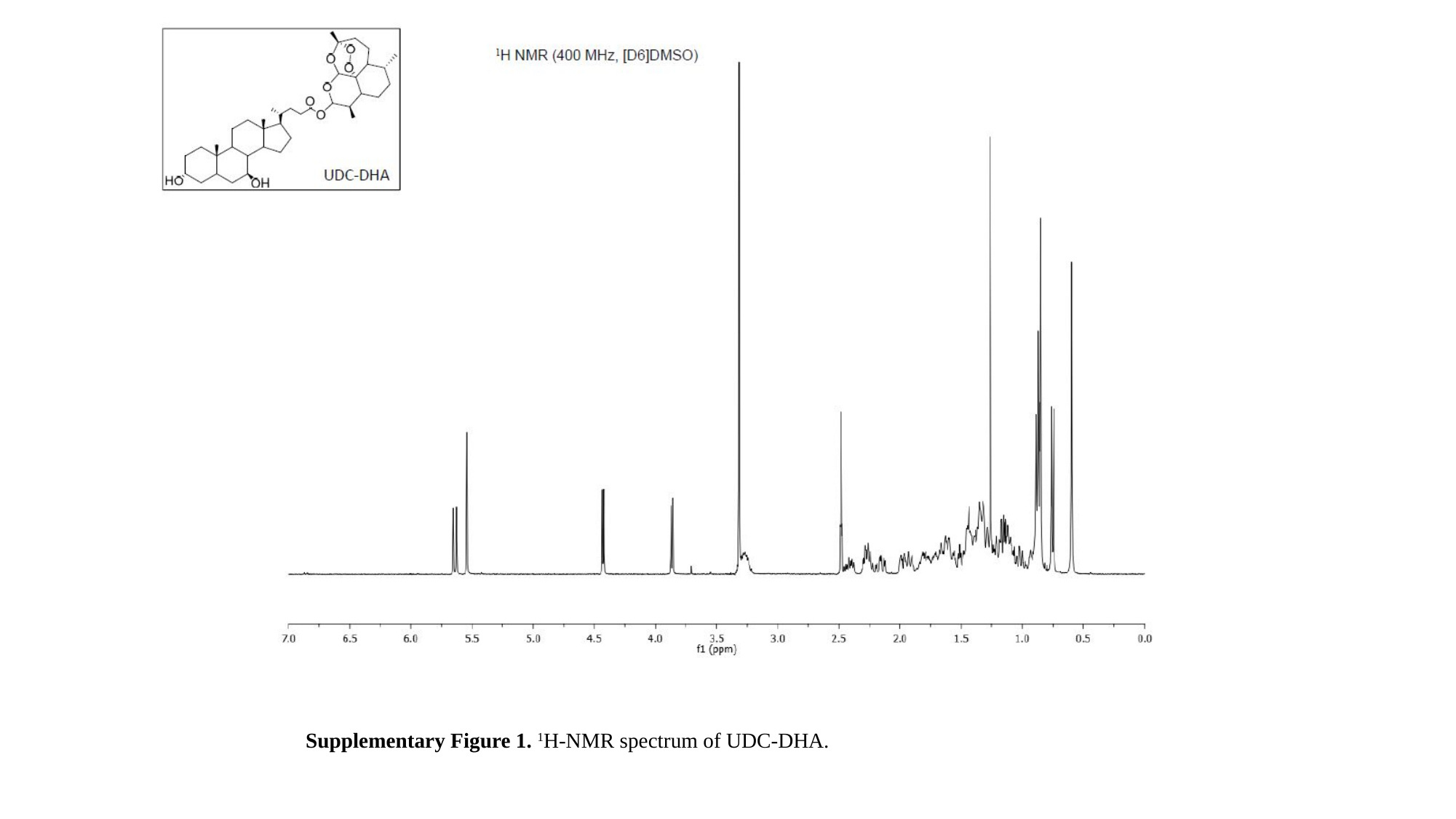

Supplementary Figure 1. 1H-NMR spectrum of UDC-DHA.

## Slide 3
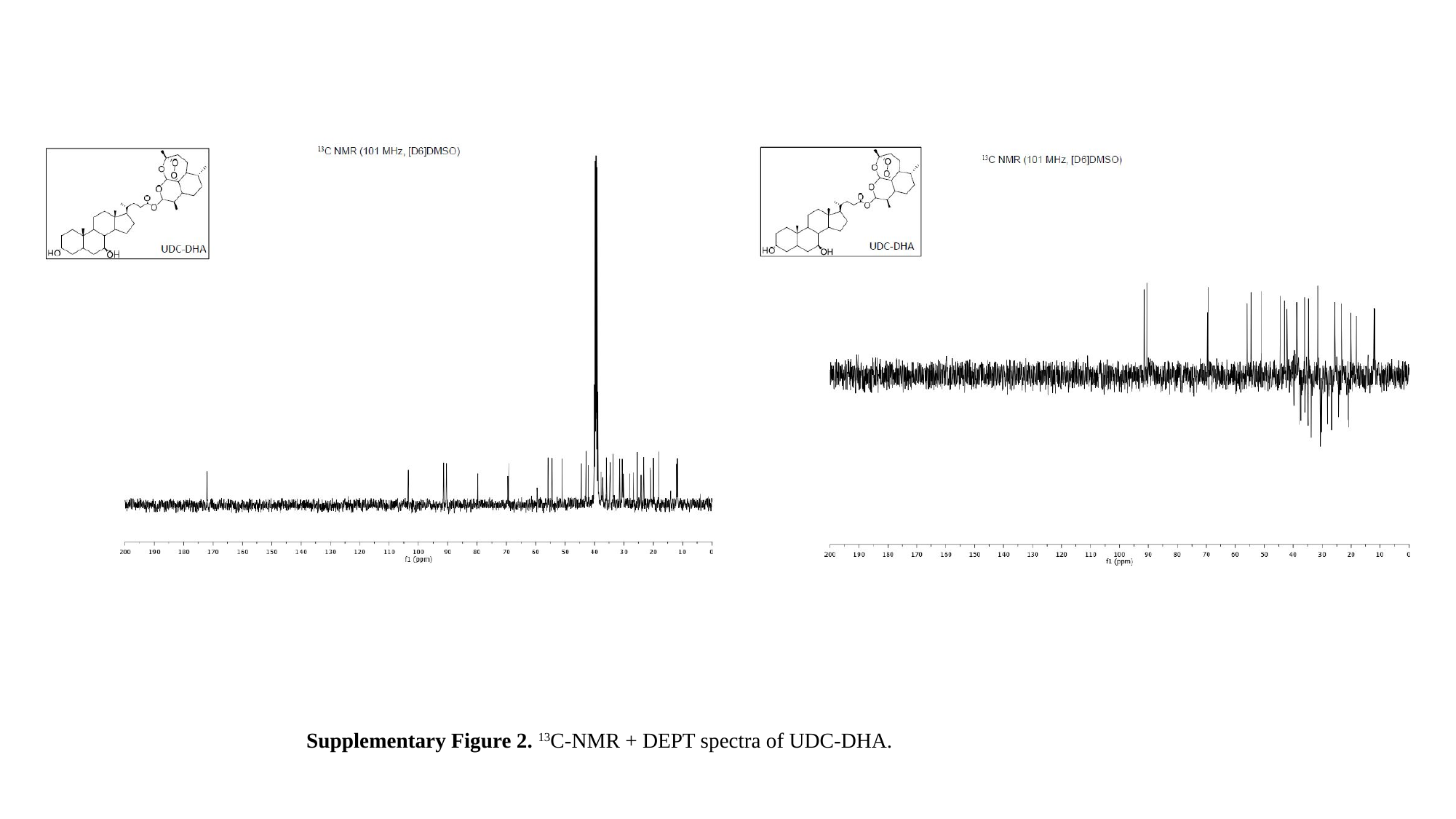

Supplementary Figure 2. 13C-NMR + DEPT spectra of UDC-DHA.

## Slide 4
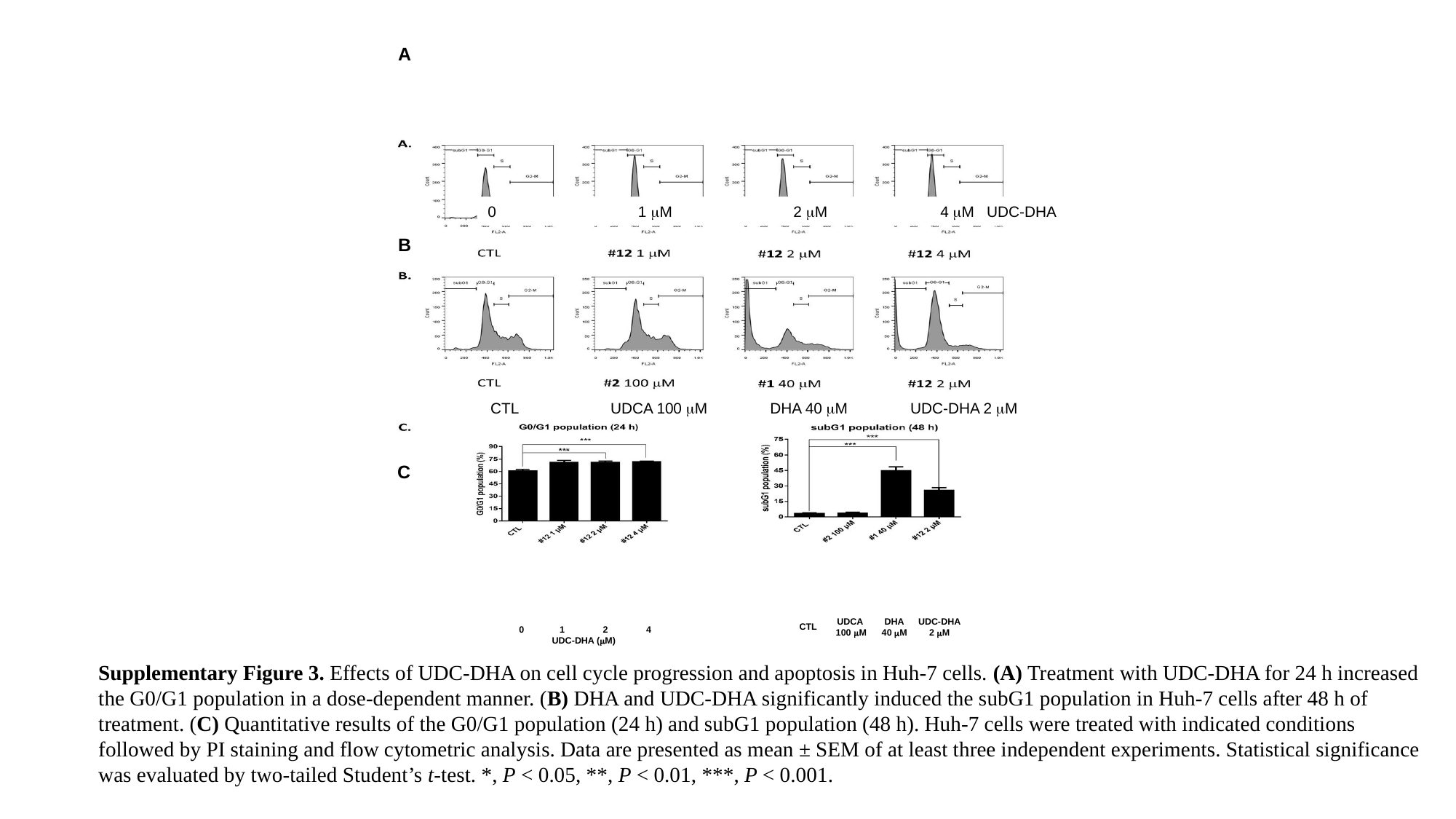

A
A
B
B
C
C
0 1 μM 2 μM 4 μM UDC-DHA
CTL UDCA 100 μM DHA 40 μM UDC-DHA 2 μM
UDCA
100 μM
DHA
40 μM
UDC-DHA
2 μM
CTL
0 1 2 4
 UDC-DHA (μM)
Supplementary Figure 3. Effects of UDC-DHA on cell cycle progression and apoptosis in Huh-7 cells. (A) Treatment with UDC-DHA for 24 h increased the G0/G1 population in a dose-dependent manner. (B) DHA and UDC-DHA significantly induced the subG1 population in Huh-7 cells after 48 h of treatment. (C) Quantitative results of the G0/G1 population (24 h) and subG1 population (48 h). Huh-7 cells were treated with indicated conditions followed by PI staining and flow cytometric analysis. Data are presented as mean ± SEM of at least three independent experiments. Statistical significance was evaluated by two‑tailed Student’s t-test. *, P < 0.05, **, P < 0.01, ***, P < 0.001.

## Slide 5
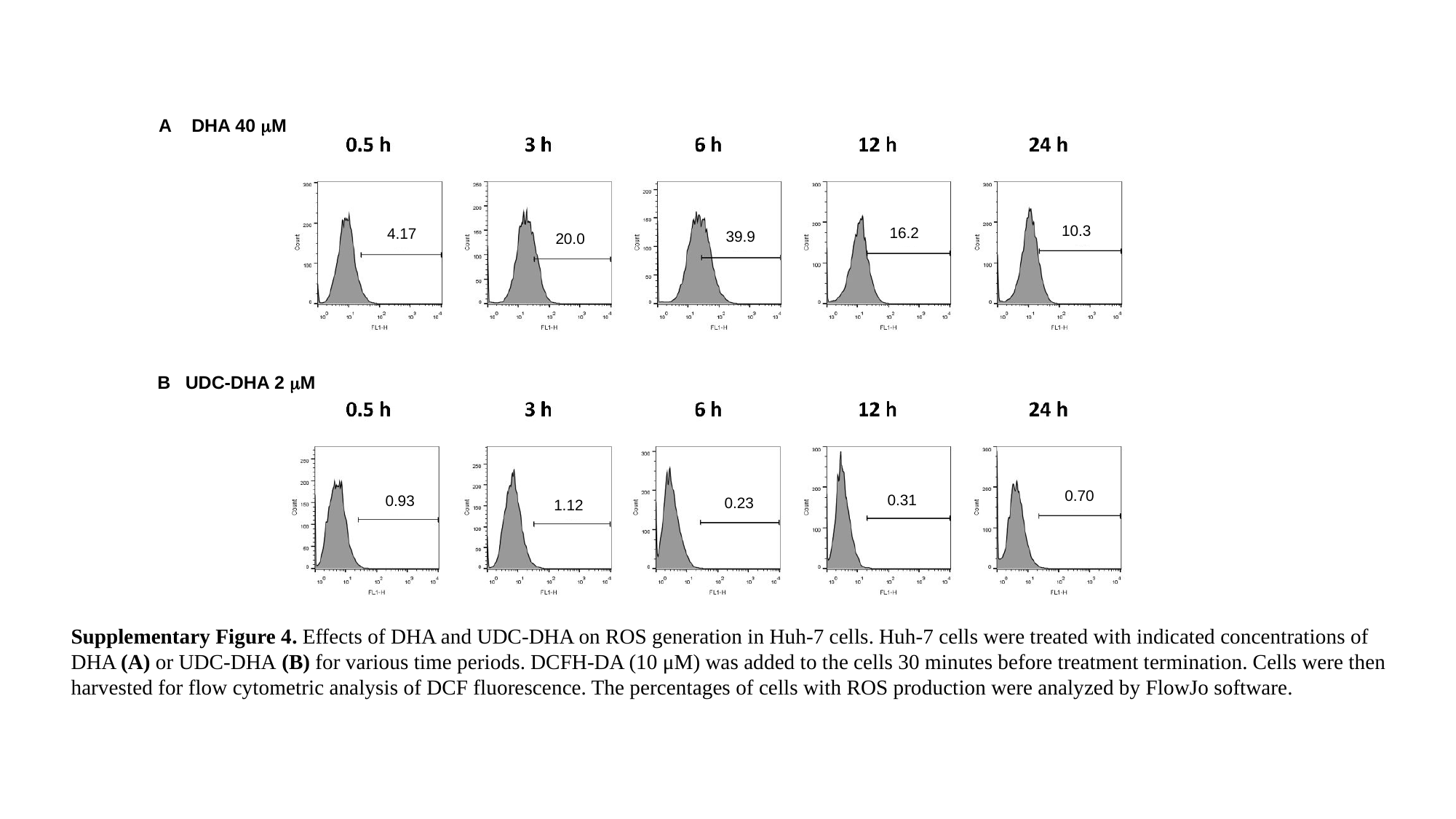

DHA 40 μM
A
10.3
16.2
4.17
39.9
20.0
B
UDC-DHA 2 μM
B
0.70
0.31
0.93
0.23
1.12
Supplementary Figure 4. Effects of DHA and UDC-DHA on ROS generation in Huh-7 cells. Huh-7 cells were treated with indicated concentrations of DHA (A) or UDC‑DHA (B) for various time periods. DCFH-DA (10 μM) was added to the cells 30 minutes before treatment termination. Cells were then harvested for flow cytometric analysis of DCF fluorescence. The percentages of cells with ROS production were analyzed by FlowJo software.

## Slide 6
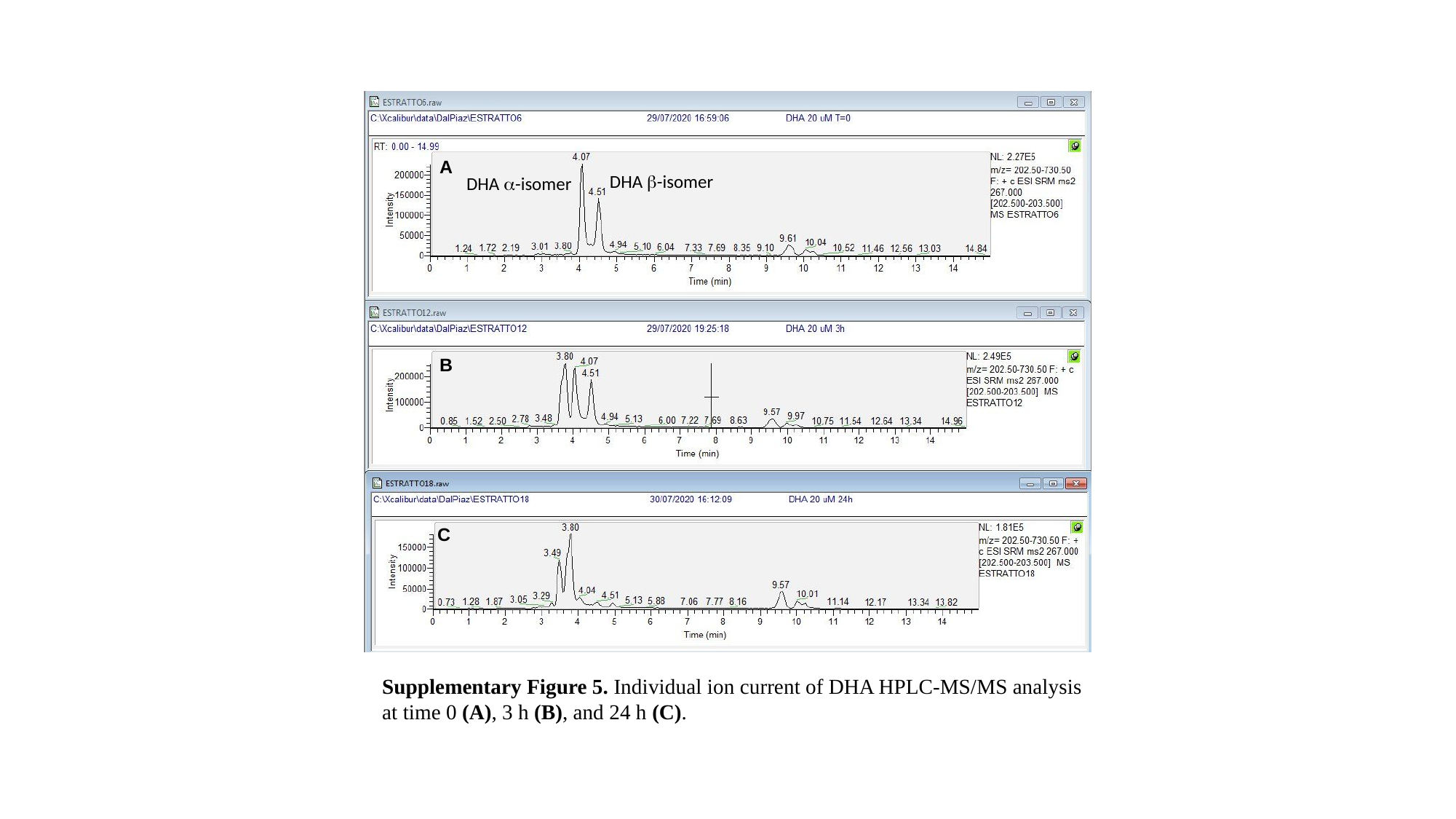

A
DHA β-isomer
DHA α-isomer
B
C
Supplementary Figure 5. Individual ion current of DHA HPLC-MS/MS analysis at time 0 (A), 3 h (B), and 24 h (C).

## Slide 7
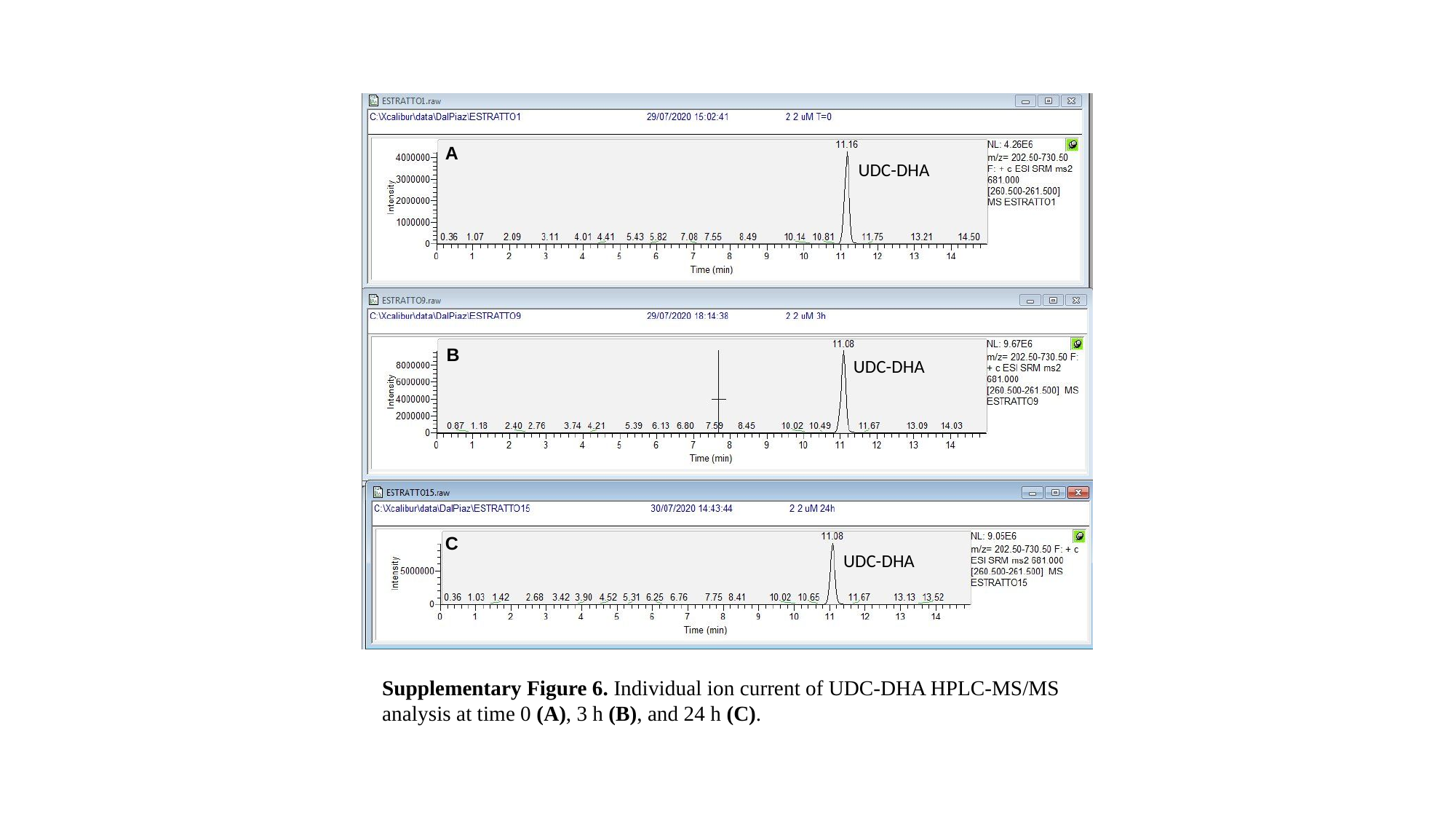

A
UDC-DHA
B
UDC-DHA
C
UDC-DHA
Supplementary Figure 6. Individual ion current of UDC-DHA HPLC-MS/MS analysis at time 0 (A), 3 h (B), and 24 h (C).

## Slide 8
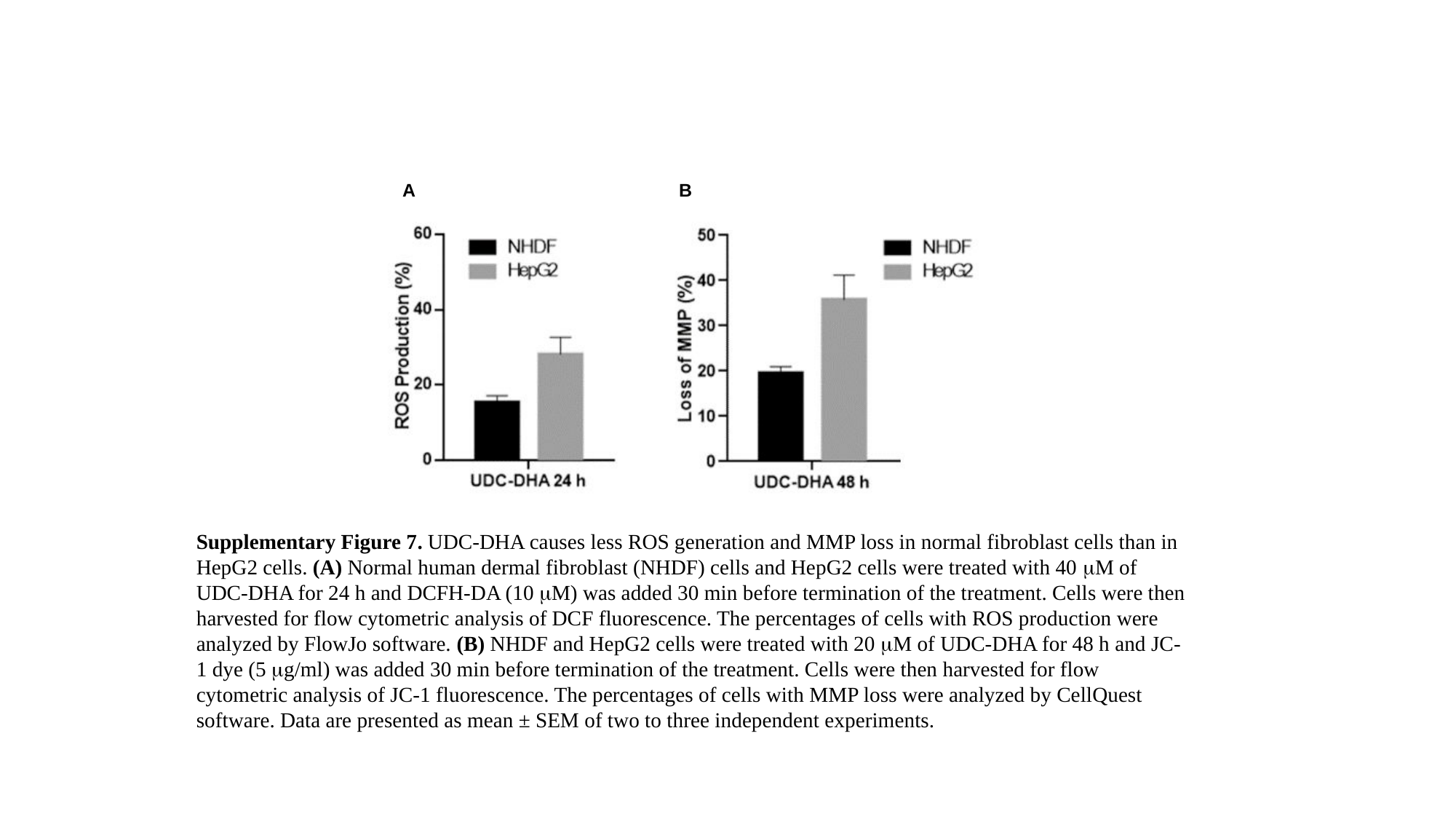

A B
Supplementary Figure 7. UDC-DHA causes less ROS generation and MMP loss in normal fibroblast cells than in HepG2 cells. (A) Normal human dermal fibroblast (NHDF) cells and HepG2 cells were treated with 40 μM of UDC-DHA for 24 h and DCFH-DA (10 μM) was added 30 min before termination of the treatment. Cells were then harvested for flow cytometric analysis of DCF fluorescence. The percentages of cells with ROS production were analyzed by FlowJo software. (B) NHDF and HepG2 cells were treated with 20 μM of UDC-DHA for 48 h and JC-1 dye (5 μg/ml) was added 30 min before termination of the treatment. Cells were then harvested for flow cytometric analysis of JC-1 fluorescence. The percentages of cells with MMP loss were analyzed by CellQuest software. Data are presented as mean ± SEM of two to three independent experiments.
